# Adipose‐specific inactivation of thyroid stimulating hormone receptors in mice modifies body weight, temperature and gene expression in adipocytes

**DOI:** 10.14814/phy2.14538

**Published:** 2020-08-18

**Authors:** Veroniqa Lundbäck, Agné Kulyté, Ingrid Dahlman, Claude Marcus

**Affiliations:** ^1^ Division of Paediatrics Department of Clinical Science, Intervention and Technology Karolinska Institutet Stockholm Sweden; ^2^ Department of Medicine Karolinska Institutet Stockholm Sweden

**Keywords:** adipogenesis, adipose‐specific knockout, BAT, body weight, thermogenesis, TSHR, uncoupling protein‐1, WAT

## Abstract

**Background:**

In obesity, the expression level of thyroid stimulating hormone receptor in adipose tissue is reduced and the levels of thyroid stimulating hormone (TSH) are often elevated within the normal range.

**Purpose/Aim:**

To investigate the role of TSHR in brown and white adipose tissue (AT) using TSHR knockout (KO) mice and the physiological phenotypes affected by the TSHR knockout.

**Methods:**

AT‐specific TSHR KO male mice and wild type (WT) controls were given a high‐fat diet (HFD) or a control diet (CD). Body weights and food consumption were recorded for 20 weeks and body temperatures for the first 3 weeks. At termination, white and brown adipocytes were isolated. Gene expressios was investigated using real‐time PCR. In a subgroup of female KO mice, glucose tolerance was investigated.

**Results:**

TSHR were partially knocked out in KO mice, which gained more weight than WT mice when fed both a CD (*p* = .03) and HFD (*p* = .003). Body temperatures were lower in KO mice on CD (*p* <.001) and on HFD (*p* <.001) than WT controls. This was in agreement with reduced gene expression of *UCP1* in brown adipocytes in the KO mice. Glucose tolerance was significantly impaired in KO mice on CD mice before termination (*p* <.01). Expression of adipogenic and lipolytic genes were reduced in KO mice, which was exacerbated by HFD. The mRNA levels of adipokines including *ADIPOQ* and *LEP* were altered in white adipocytes of KO mice.

**Conclusions:**

TSHR KO led to dysfunction of both white and brown AT and predisposition to excess body weight gain in mice. Our data show that TSHR in AT regulates glucose tolerance, lipid metabolism, adipokine profile, and thermogenesis.

## Graphical abstract

TSHR in adipose tissue appears to be important in regulating body composition in mice, separately from the effects of circulating thyroid hormone levels. A partial deletion of TSHR in adipocytes results in an increased body weight and an altered adipocyte‐specific gene expression in brown and white adipocytes.

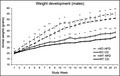

## 1 INTRODUCTION

Thyroid stimulating hormone (TSH) is a hormone released by the pituitary gland. It primarily regulates thyroid hormone homeostasis by activating G‐protein coupled TSH receptors (TSHR) on the thyroid gland (Oppenheimer, 1979). Beyond the thyroid gland, TSHR is expressed in several other tissues, including adipose tissue (AT) in both humans and rodents (Haraguchi, Shimura, Shimura, Lin, Endo, & Onaya, 1996; Janson, Rawet, Rawet, Perbeck, & Marcus, 1998), where it is involved in multiple processes important for AT function. TSHR mediates the lipolytic effects of TSH in white adipocytes of human neonates (Marcus, Ehren, Ehren, Bolme, & Arner, 1988). We have previously reported that TSHR is important for white adipocyte growth in mice (Elgadi, Zemack, Zemack, Marcus, & Norgren, 2010) and recent studies indicate a possible role of TSHR signaling in adipocyte differentiation (Lu & Lin, 2008) and lipolysis in mice (Endo & Kobayashi, 2012).

Brown adipose tissue (BAT) is responsible for nonshivering thermogenesis mediated by the BAT‐specific protein uncoupling protein‐1 (UCP1; Cannon & Nedergaard, 2004; Nedergaard et al., 2001). BAT is of physiological significance in cold‐acclimated and hibernating mammals, and possibly in human infants at birth, for maintaining body temperature (Aherne & Hull, 1966; Cannon & Nedergaard, 2004). Recent studies have reported BAT to be active and functional also in adult humans (Nedergaard, Bengtsson, Bengtsson, & Cannon, 2007; Virtanen et al., 2009), although the physiological significance remains uncertain. Previous studies of mutant hyt/hyt mice (TSHR^hyt/hyt^) that are hypothyroid due to hyt, a mutation in the TSHR, indicated that not only thyroid hormone but also TSH is involved in the regulation of UCP1 in mouse BAT (Endo & Kobayashi, 2008).

Although TSHR has been implicated in the regulation of specific adipocyte functions, the mechanistic role of TSHR in these cells is unclear, as are the potential target genes. Mouse strains that lack either functional TSH or TSHR display hypothyroidism with thyroid hyperplasia. These mice exhibit dysfunctional TSH or TSHR signaling in all body tissues, and it is therefore not possible in these strains to study the direct role of TSHR in a specific tissue, such as the AT. Thus, in order to study the role of TSHRs in adipocyte function we generated a mouse model with normal thyroid function but with TSHR specifically down‐regulated in AT (Elgadi et al., 2010). We examined whether adipose‐specific inactivation of TSHR, affects body weight and temperature, glucose tolerance, and expression of genes central to adipogenesis and adipocyte function, thereby perturbing overall adipose tissue physiology, and whether these effects could be aggravated in animals fed with a high‐fat diet.

## 2 RESEARCH DESIGN AND METHODS

### 2.1 Animals

The generation of the adipose‐specific TSHR (TSHRloxP/loxP Cre) knockout (KO) mouse strain bred on a C57Bl6 background has previously been described (Elgadi et al., 2010). In brief, Cre‐mice expressing the Cre‐recombinase under the control of the Fabp4/ap2‐promoter (B6.Cg‐Tg (Fabp4‐Cre) 1Rev/J stock no. 005069 Jax Laboratory, Maine, US) were bred with mice in which the LoxP‐sites had been inserted into exon 10 of the TSHR gene, which encodes the transmembrane β‐subunit of the protein. The KO mice were continuously bred and maintained at the Preclinical Laboratory animal facility at the Karolinska Institutet (PKL, Karolinska Institutet, Stockholm, Sweden). All animals in the study, TSHR KO together with corresponding C57Bl6 wild type (WT) mice were bred at the facility. The mice were maintained under standard conditions of a 12‐hr light/dark cycle at an environmental temperature of 20–22°C and fed ad libitum according to the regulations of the Federation of European Laboratory Animal Science Association.

The TSHR KO genotype and presence of Cre‐recombinase transgene were determined from DNA extracted from ear skin biopsy with KAPA Express Extract Kit followed by PCR using KAPA2G Fast Genotyping Mix (KAPA Mouse Genotyping Kit, KAPA Biosystems, Sigma Aldrich). For the detection of Cre, the primer sequences used were: 5’‐GCGGTCTGGCAGTAAAAACTATC and 3’‐GTGAAACAGCATTGCTGTCACTT. For the determination of TSHR KO, the primer sequences used were: 5’‐ATAAGACAGCAAAGCTGGTTTGT and 3’‐ATGAGCAGGACGAAGATATTGC.

The study protocol was approved by the Stockholm South Animal Board Ethics Committee (D.no S65–13, D7‐16).

### 2.2 Dietary intervention

At 4–5 weeks of age, male animals were divided into four groups (*n* = 8–10 per group) according to genotype and diet. KO and WT mice received either a high‐fat diet (HFD) or matched control diet (CD) (Research Diets Inc. D12492 (60% kcal from fat) and D12450J (10% kcal from fat) with matching sucrose)) ad libitum. Food consumption was measured manually as grams consumed per cage, and was monitored once weekly for 20 weeks. Calories (kcal) consumed per cage and total kcal consumed during the study period were calculated.

### 2.3 Body weight record and temperature measurement

Body weights of male mice were measured once weekly for 20 weeks. Body temperatures were measures once weekly during the first 3 study weeks in all male mice. Measurements were performed in the morning at an environmental temperature of 20–22°C using a rectal probe attached to a digital readout thermocouple (BAT‐12, Microprobe Thermometer, Physitemp Instruments, Inc, NJ, USA) with a resolution of 0.1°C and accuracy of ±0.1°C.

### 2.4 AT collection and isolation of adipocytes

After 20 weeks, all male mice were sedated using isoflurane and thereafter euthanized by cervical dislocation. Interscapular BAT depots and epididymal WAT depots were collected and placed in 0.9% saline whereafter adipocytes were isolated using the collagenase method as described (Rodbell, 1964). In brief, adipose tissue was cut into fragments and isolated from stroma by incubation with collagenase type I (Sigma, St. Louis, MO, USA) for 1 hr at 37°C in Krebs–Ringer phosphate buffer, pH 7.4, containing 40 g/L of bovine albumin. The samples were washed in Krebs–Ringer phosphate buffer, pH 7.4, and aggregated material was removed by filtration through a silk cloth. Adipocytes were kept frozen at −80°C until RNA extraction.

### 2.5 Oral glucose tolerance test

Glucose tolerance was investigated in 8–9‐week‐old female KO mice and corresponding WT mice ( *n* = 6–8 per group) by baseline oral glucose tolerance test (OGTT). The test was repeated after the mice had received either HFD or CD for 10 weeks. Pretest body weights were measured and animals were then deprived of food for 5 hr. They orally received a 20% aqueous glucose solution (Glucose, APL Pharma Specials, Sweden) at a dose of 1.5 grams of glucose per kilogram of body weight. Blood was drawn from the tail vein before glucose administration and at 15, 30, 60, 90 and 120 min after dose. Venous plasma glucose was measured in each blood sample using a glucometer (HemoCue system 201+, Hemocue AB Ängelholm, Sweden). Body weights were monitored weekly to ensure the health of the mice.

### 2.6 RNA extraction and Real‐time PCR

Total RNA was isolated from epididymal white and interscapular brown adipocytes using the Qiagen lipid tissue kit (Cat no. 74804, Qiagen). cDNA was generated using 1 μg aliquots of the RNA (iScript, Thermo Scientific). The expression of specific mRNA was determined using real‐time PCR (CFX thermocycler, BioRad) using Taqman assays (Life Technologies) for *TSHR* (Mm01337707_m1), glyceraldehyde 3‐phosphate dehydrogenase (*GAPDH* Mm99999915_g1), uncoupling protein‐1 (*UCP1* m00494069_m1), peroxisome proliferator‐receptor gamma (*PPARG* Mm00440940_m1), CCAAT/enhancer‐binding protein alpha (*CEBPA* Mm00514283_s1), hormone‐sensitive lipase (*LIPE* Mm00495359_m1), beta‐1adrenergic receptor (*ADRB1* Mm00431701_s1), beta‐3 adrenergic receptor (*ADRB3* Mm00442669_m1), solute carrier family 2/glucose transporter type 4 (*SLC2A4* Mm00436615_m1), insulin receptor substrate 1 (*IRS1* Mm01278327_m1), adiponectin (*ADIPOQ* Mm00456425_m1), and leptin (*LEP* Mm00434759_m1). All gene expression assays were normalized to *GAPDH* expression. Relative gene expressions were calculated using the delta‐delta Ct method (Livak & Schmittgen, 2001). For each experimental group (*n* = 6–8), expression levels were expressed as a fold‐change compared to WT mice fed on CD.

### 2.7 Serum ELISA

Adipokines including adiponectin and leptin in serum were analyzed using commercially available ELISA kits (Mouse adiponectin ELISA kit, Invitrogen and Mouse Leptin ELISA kit, Invitrogen).

### 2.8 Statistical analysis

Statistical software JMP 13.0.0 was used for analysis. Results are expressed as means ± standard error of mean (*SEM*) or 95% confidence intervals (95% CI). Between group comparisons were performed using independent *t*‐test or one‐way ANOVA with Tukey–Kramers post hoc test. *p* <.05 was considered significant.

## 3 RESULTS

### 3.1 Expression of TSHR in adipocytes

**FIGURE 1:**
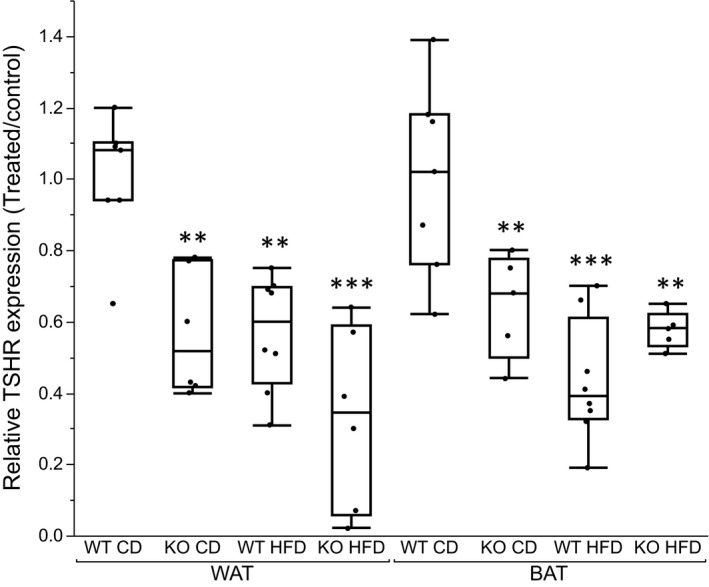
Relatvie gene expression of *TSHR* in isolated adipocytes from white (WAT) and brown (BAT) adipose tissue. Box plots represent difference in gene expression of groups relative to the WT mice on control diet. Between group comparisons with One‐way ANOVA and Tukey–Kramer post hoc test. (*n* = 6–8). *p <.05, **p <.01, ***p <.001 represent significant differences between treated groups (KO CD, KO HFD and WT HFD) and WT CD group. ^#^ *p* <.05, ^##^ *p* <.01, ^###^ *p* <.001 represent significant difference between WT HFD and KO HFD groups

In isolated white and brown adipocytes from KO mice, the expression of *TSHR* was down‐regulated by approximately 40% as compared to WT mice (Figure 1). HFD reduced *TSHR* expression in WT animals to the same extent as KO on CD. In KO mice, feeding of HFD further decreased the *TSHR* expression in white adipocytes but not in brown adipocytes.

### 3.2 Weight development and calorie intake

**FIGURE 2:**
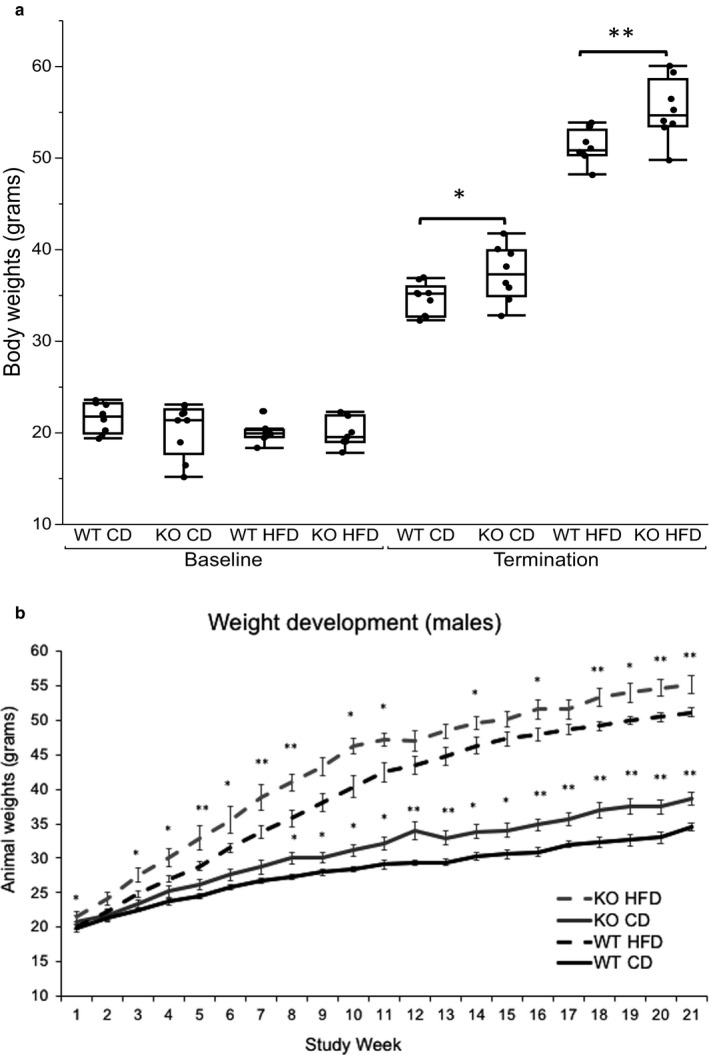
(a) Body weight before study start and after 20 weeks before termination. Between group comparisons at baseline with One‐way ANOVA and Tukey–Kramer posthoc test. At termination independent *t*‐test was used between WT CD and KO CD and between WT HFD and KO HFD. *p <.05, **p <.01, ***p <.001. (b) Body weight development during 20 weeks. Data are presented as mean weight per group and per week (grams). Error bars represent standard error of mean. Between group comparisons with Independent *t*‐test: WT CD versus KO CD and WT HFD versus KO HFD, respectively. *p <.05, **p <.01, ***p <.001

In KO and WT mice, body weight development was followed for 20 weeks in a dietary intervention. Body weights did not differ significantly between KO mice and WT mice at baseline, i.e., at 4–5 weeks of age (Figure 2a). At termination KO mice weighted more than their respective control (mean difference in weight of CD fed mice 2.8 ± 1.25g and HFD fed mice 4.1 ± 1.28g), (Figure 2a). Fed with CD, KO mice became significantly heavier from study week 8 and continued to increase in weight faster than WT throughout the study period. KO mice on HFD already had a greater body weight at study week 4 that persisted until the end of the study period, but after 11 weeks the growth rate was similar for WT and KO mice (Figure 2b).

**FIGURE 3:**
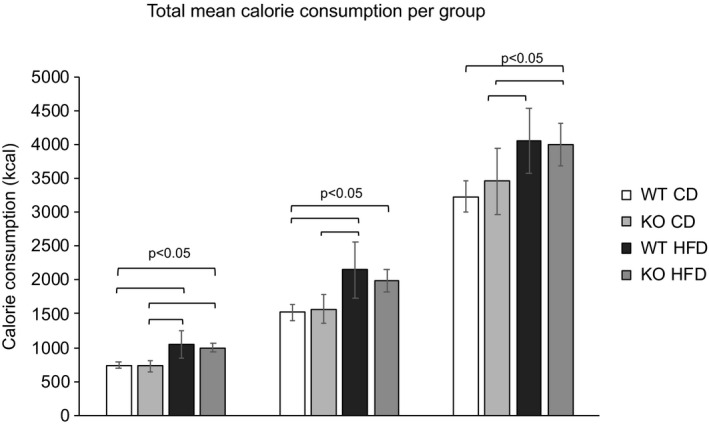
Total mean calorie consumption per group (*n* = 4) at 5, 10 and 20 weeks. Data are presented as mean kcal per group. Error bars represent 95% confidence intervals. Comparison of means with One‐way ANOVA and Tukey–Kramer post hoc test

Food consumption was measured weekly for 20 weeks as grams per cage (each cage housed two mice) and converted to kcal consumed per week and combined into total kcal for 5, 10 and 20 weeks. Total calorie intake was higer in HFD groups compared to CD groups. There were no significant differences in total calorie intake between mouse groups fed with the same diet (Figure 3).

### 3.3 Regulation of glucose metabolism

**FIGURE 4:**
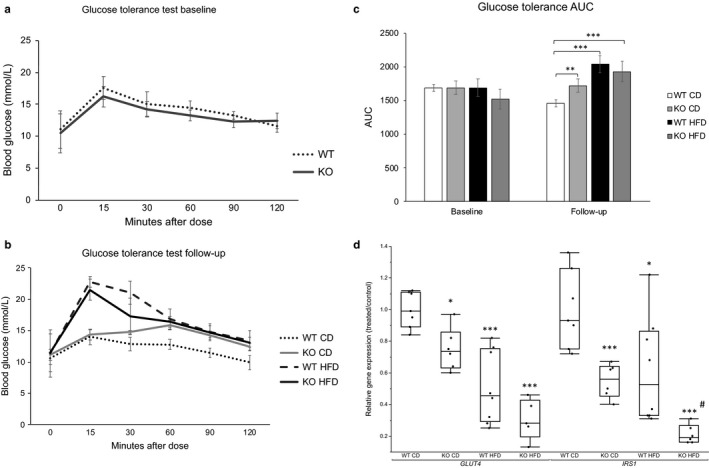
(a) Blood glucose response over time (0–120 min) to an orally administered glucose solution at baseline before the start of the diet intervention. Data are presented as blood glucose (mmol/L). Error bars represent 95% Confidence intervals. (b) Blood glucose response over time (0–120 min) to an orally administered glucose solution at follow‐up, after receiving CD of HFD for 10 weeks. Data are presented as blood glucose (mmol/L). Error bars represent 95% Confidence intervals. (c) Bars represent the area under the curve from an oral glucose tolerance test at baseline and after 10 weeks on CD or HFD (*n* = 6–8). Data are presented as total mmol/L glucose in blood. Error bars represent 95% Confidence intervals. Between group comparisons with One‐way ANOVA and Tukey–Kramer posthoc test. *p <.05, **p <.01, ***p <.001. (d) Relative gene expression of genes central to glucose uptake and insulin signaling (*GLUT4* and *IRS1*) in white isolated adipocytes. Box plots represent gene expression of groups relative to the WT mice on CD. Between group comparisons with One‐way ANOVA and Tukey–Kramer post hoc test. (*n* = 6–8). *p <.05, **p <.01, ***p <.001 represent significant differences between treated groups (KO CD, KO HFD and WT HFD) and WT CD group. ^#^ *p* <.05, ^##^ *p* <.01, ^###^ *p* <.001 represent significant difference between WT HFD and KO HFD groups

Female mice underwent an OGTT at 8 weeks of age (baseline; Figure 4a) and after receiving either the CD or the HFD for 10 weeks (follow‐up; Figure 4b). At baseline, we found no differences in glucose tolerance between KO and WT mice. At follow‐up, KO mice on CD had a higher area under the curve (AUC) compared to corresponding WT mice (Figure 4c). On HFD, both WT and KO mice showed signs of insulin resistance with higher AUC than CD fed mice. In line with the observed glucose tolerance, mRNA expression in white adipocytes of *SLC2A4* and *IRS1* in white adipocytes was reduced in all groups compared to control WT mice on CD (Figure 4d).

### 3.4 Body temperatures and brown adipocyte gene expression

**FIGURE 5:**
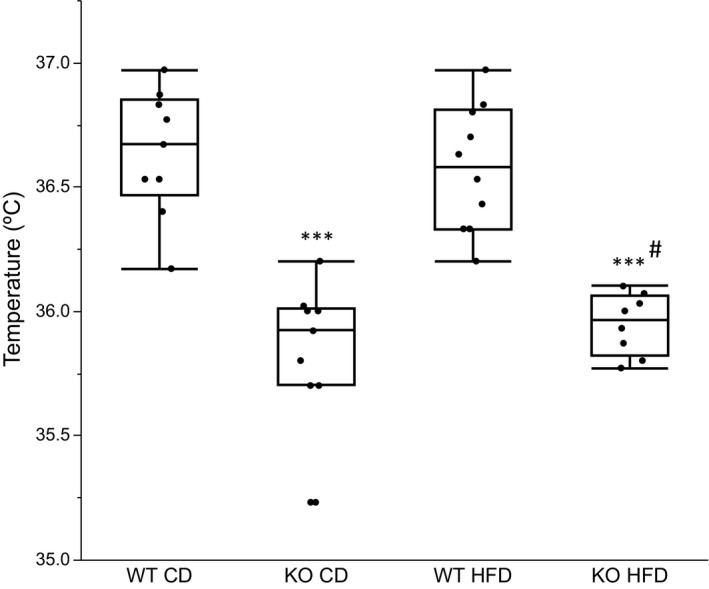
Mouse body temperatures represented by box plots with individual data points representing the mean of temperatures measured once weekly the first three study weeks. Between group comparisons with One‐way ANOVA and Tukey–Kramer post hoc test. (*n* = 6–8). *p <.05, **p <.01, ***p <.001 represent significant differences between treated groups (KO CD, KO HFD and WT HFD) and WT CD group. ^#^ *p* <.05, ^##^ *p* <.01, ^###^ *p* <.001 represent significant difference between WT HFD and KO HFD groups

**FIGURE 6:**
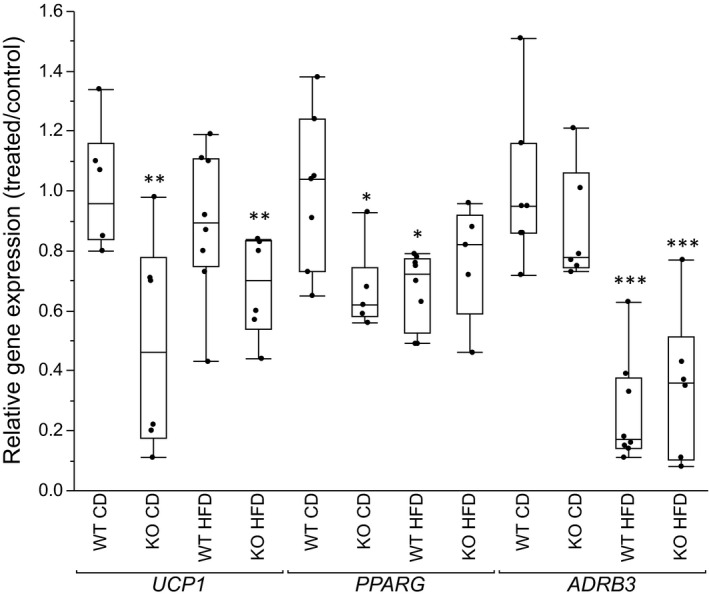
Relative gene expression in brown isolated adipocytes of genes central to brown adipocyte function (*UCP1, PPARG* and *ADRB3)*. Box plots represent difference in gene expression of groups relative to the WT mice on control diet. Between group comparisons with One‐way ANOVA. *p <.05, **p <.01, ***p <.001 represent significant differences between treated groups (KO CD, KO HFD and WT HFD) and WT CD group. ^#^ *p* <.05, ^##^ *p* <.01, ^###^ *p* <.001 represent significant difference between WT HFD and KO HFD groups

Body temperatures were measured once weekly during the first 3 weeks of the study period and mean temperatures were compared (Figure 5). KO mice had a lower body temperature compared to WT mice on both CD and HFD. For KO mice on CD, body temperature continued to fall during the initial study weeks, whereas the temperature increased for KO on HFD during week 3 (data not shown). At the end of the study (week 20), the expression of key genes controlling BAT function was measured (Figure 6). We observed a reduction in brown adipocyte *UCP1* expression in both KO groups. Furthermore, the expression of *PPARG*, also known as the master regulator of adipogenesis, was down‐regulated in KO mice on CD. Expression of the beta adrenoreceptor *ADRB3*, involved in induction of themorgenesis, was not different between KO and WT mice on CD, but decreased by HFD in both KO and WT mice.

### 3.5 White adipocyte gene expression

**FIGURE 7:**
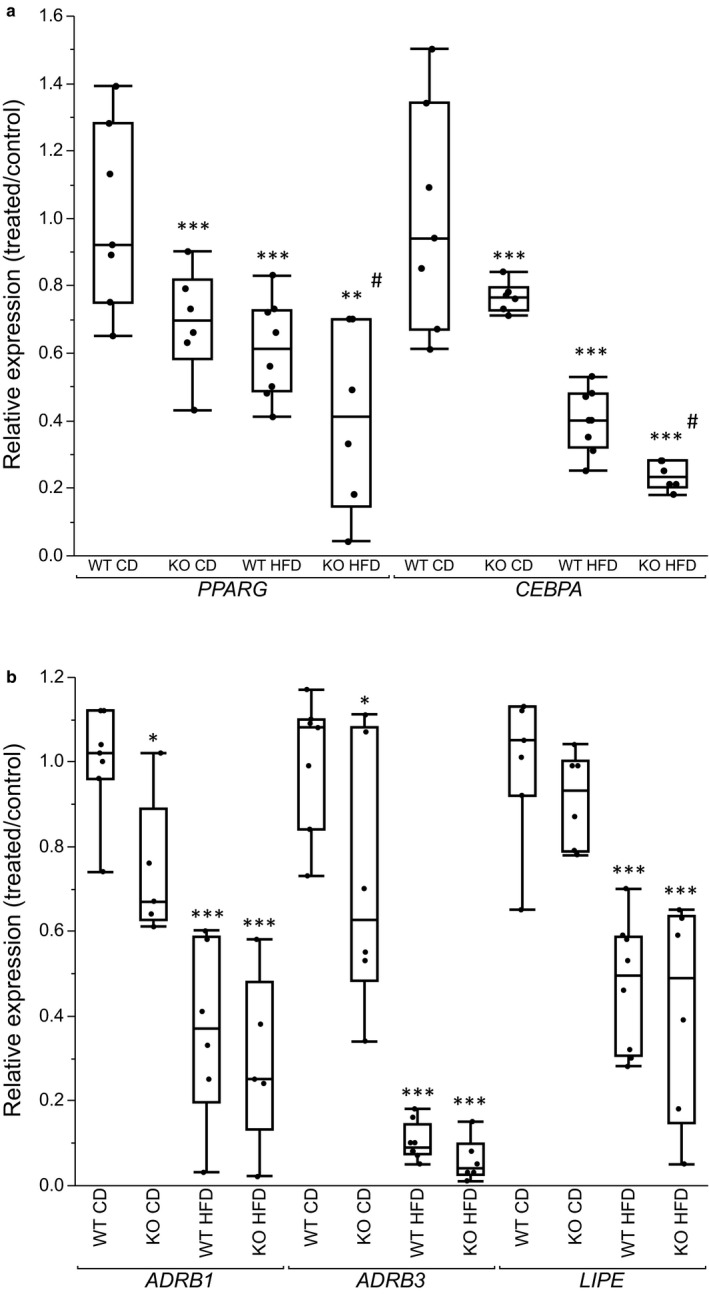
(a,b) Gene expression in isolated white adipocytes; (a) *PPARG, and CEBPA*. (b) *ADRBR1, ADRB3* and *LIPE*. Box plots represent gene expression of groups relative to the WT mice on CD. Between group comparisons with One‐way ANOVA and Tukey–Kramer post hoc test. (*n* = 6–8). *p <.05, **p <.01, ***p <.001 represent significant difference between treated groups (KO CD, KO HFD and WT HFD) and WT CD group. ^#^ *p* <.05, ^##^ *p* <.01, ^###^ *p* <.001 represent significant difference between WT HFD and KO HFD groups

TSHR has been implicated in regulation of adipogenesis and lipolysis (Endo & Kobayashi, 2012; Lu & Lin, 2008). We studied the gene expression of genes regulating these processes in order to elucidate whether TSHR may transcriptionally influence adipogenesis and lipolysis. We observed a down‐regulation of *PPARG* and *CEBPA* in KO mice on CD (Figure 7a). The expression was further reduced in HFD groups, and was significantly lower in HFD KO mice compared to HFD WT mice. For genes involved in lipolysis, we observed a reduction in the expression of *LIPE* in both KO and WT mice on HFD (Figure 7b). Expression of adrenergic receptors *ADRB1* and *ADRB3* was reduced in KO mice on CD and in both HFD groups (Figure 7b).

### 3.6 Adipokine mRNA and serum protein levels

**FIGURE 8:**
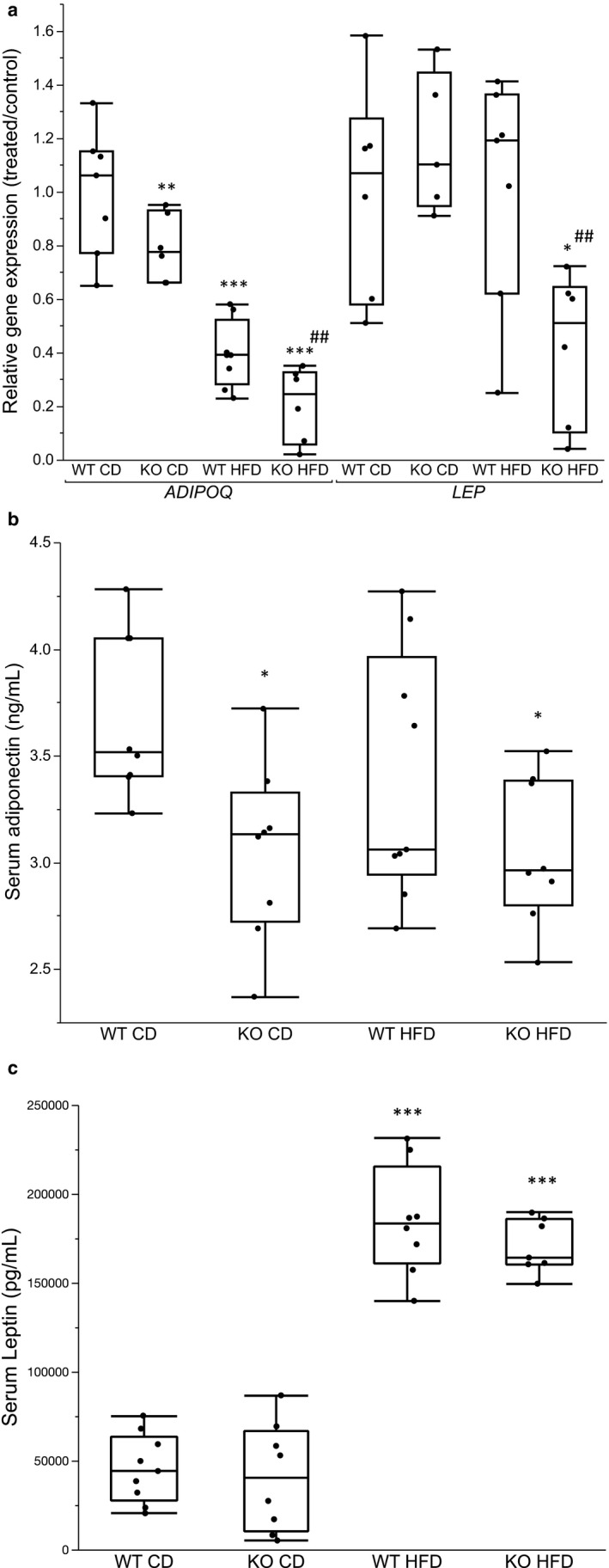
(a) Gene expression in isolated white adipocytes of *ADIPOQ* and *LEP*. Box plot represents difference in gene expression of groups relative to the WT mice on CD. Between group comparisons with One‐way ANOVA and Tukey–Kramer post hoc test. (*n* = 6–8). *p <.05, **p <.01, ***p <.001 represent significant difference between treated groups (KO CD, KO HFD and WT HFD) and WT CD group. ^#^ *p* <.05, ^##^ *p* <.01, ^###^ *p* <.001 represent significant difference between WT HFD and KO HFD groups. (b,c) Box plots represent serum levels of (b) adiponectin (ng/mL) and (c) Leptin (pg/mL). Between group comparisons with One‐way ANOVA and Tukey–Kramer post hoc test. (*n* = 6–8). *p <.05, **p <.01, ***p <.001 represent significant difference between treated groups (KO CD, KO HFD and WT HFD) and WT CD group. ^#^ *p* <.05, ^##^ *p* <.01, ^###^ *p* <.001 represent significant difference between WT HFD and KO HFD groups

The expression of *ADIPOQ* was down‐regulated in KO mice on CD and further reduced in HFD groups, whereas *LEP* expression was markedly reduced in HFD KO mice with no observed change in the other groups (Figure 8a). In serum, adiponectin levels were lower only in KO mice (Figure 8b). Leptin levels were markedly elevated in HFD groups, both KO and WT, as expected but there were no differences between KO mice and their corresponding WT controls on their respective diets (Figure 8c).

## 4 DISCUSSION

In this study, we observed that TSHR in brown and white adipose tissue is involved in body weight regulation in mice both on CD and HFD. Animals partially lacking functional TSHR in AT were more susceptible to developing obesity compared to corresponding WT mice. This is attributable to disturbances in nonshivering thermogenesis and adipocyte metabolism.

It has been previously reported that knocking down TSHR in mouse 3T3‐L1 preadipocytes resulted in delayed cell differentiation, suggesting that TSHR has a regulatory role in adipogenesis (Lu et al., 2012). Another study in the same mouse strain as used in our study, showed that partially knocking out TSHR in AT resulted in a greater adipocyte volume, strengthening the evidence of a potential regulatory role for TSHR in adipocyte function (Elgadi et al., 2010). TSHR KO mice have decreased expression of genes with central functions in adipogenesis (PPARG and CEBPA; Rosen et al., 2002). The reduction in mRNA levels of both PPARG and CEBPA observed in this study is in line with previous observations of a reduced adipogenesis observed in cell culture where TSHR was inactivated (Haraguchi et al., 1999; Lu et al., 2012).

Knocking out *TSHR* has been shown to affect the expression of markers for insulin sensitivity in white adipocytes (Shepherd & Kahn, 1999; Sun et al., 1992). A proper insulin response in adipocytes requires coordinated action of multiple players along the signaling cascade, including *IRS1*, which encodes for a mediator of insulin signaling, *SLC2A4*, encoding the glucose transporter GLUT4, and *ADIPOQ*, encoding the insulin‐sensitizing hormone adiponectin. These genes were all down‐regulated in KO mice in this study. Furthermore, the serum level of adiponectin was reduced indicating a direct effect of TSHR on the adiponetin production. These findings indicate a direct negative impact of down‐regulating TSHR on insulin sensitivity in adipocytes, which might be secondary to an impaired adipogenesis.

Furthermore, the gene expression of lipolytic genes was reduced by the knockout. We observed a reduction in the expression of *ADRB1* and *ADRB3*, both G‐protein coupled receptors inducing lipolysis when activated by catecholamines (Arner, 2005). A reduction in gene expression of the major lipolysis‐regulating catecholamine receptors could result in an impaired ability to stimulate the hydrolysis of lipids (lipolysis) in adipocytes (Jenkins‐Kruchten et al., 2003; Ryden et al., 2007). The expression of *LIPE*, a gene encoding for hormone‐sensitive lipase, a rate‐limiting enzyme in lipolysis, was also reduced in our model. These, findings implicate a lipolytic role for TSHR by enhancing adrenergic activity in adipocytes, in addition to the cannonical TSH‐TSHR signaling cascade. Taken together, adipocyte dysfunction due to impaired adipogenesis and reduced lipid mobilization could result in retention of lipids and contribute to AT expansion and weight gain.

The expression of LEP, encoding for the adipokine leptin, was down‐regulated in KO mice on HFD but not in WT animals on HFD. This may indicate a direct effect of TSHR on LEP expression which is implicated in a previous study that demonstrated a direct stimulatory effect of TSH on leptin secretion in adipocytes (Menendez et al., 2003). However, as HFD also caused a down‐regulation of *TSHR* in adipocytes the causality is difficult to establish. The observed increase of leptin in serum is likely a result of the obese state in both WT and KO on HFD.

During the first 3 weeks of the study, TSHR KO mice had a reduced body temperature and also a down‐regulated *UCP1* expression in BAT. It is well known that β3 adrenergic receptors have a central role in thermoregulation and energy homeostasis through their mediation in sympathetic stimulation of BAT thermogenesis (Silva, 2005). We found that *ADRB3* expression was unaffected in KO mice on CD pointing toward a direct regulatory role for TSHR‐signalling on UCP1 activity. The lower body tempretature may therefore be due to reduced UCP1 activity which in turn resulted in a lower body temperature and potentially reduced energy consumption. We did not observe any significant differences in total calorie intake between KO and corresponding WT groups despite marked differences in weight gain. Nevertheless, these data together show that the observed weight gain in the adipose‐specific TSHR KO mice was due to reduced effects of TSHR in both WAT and BAT.

The down‐regulation of TSHR expression in adipocytes of WT animals fed with HFD was substantial, such to the same extent as in KO mice on CD. Given the notable detrimental effects on metabolism observed in our KO model, these data suggest that TSHR down‐regulation in obesity may contribute in the manifestation of a range of metabolic abnormalities, including glucose intolerance, altered adipokine profile, and dysfunctional thermogenesis. Future investigation is warranted to further elucidate the underlying mechanisms in detail.

## 5 LIMITATIONS

The mouse model used in our study is a conditional knockout mouse created using the Fabp4/aP2‐Cre‐lox system. More recent studies have shown that the Fabp4/aP2‐Cre is not as effective or specific as for example the adiponectin‐Cre‐lox system (Jeffery et al., 2014). This explains the partial knockout of TSHR observed where the mRNA expression of TSHRs in AT varied between AT depots due to the Cre‐recombinase not being expressed in all adipocytes. In addition, the effect of TSHR KO in macrophages, which also exhibit aP2 promoter activity, cannot be determined. Nevertheless, our conditional KO model is still more robust than whole‐body KO mice in studying the metabolic effects of TSHR in AT. Besides, our results on isolated adipocytes from WAT and BAT clearly demonstrated the adipocyte‐specific effects of TSHR. Some of our experiments were performed in only one sex owing to animal availability. It is unknown whether TSHR KO effects are sex‐dependent, and future studies are warranted to comprehensively investigate such effects in our model.

## 6 CONCLUSIONS

In this study, we demonstrated the role of TSHR in AT in regulating metabolism in mice, independent of the circulating thyroid hormone levels. A partial deletion of TSHR in adipocytes results in an increased body weight, a reduced expression of lipolytic genes and an altered adipokine profile in brown and white adipocytes. This results in white and brown adipocyte dysfunction and difficulties in coping with overfeeding and obesity. TSHR is required in mice for maintaining a normal body temperature, indicating that TSHR in BAT are important for thermogenesis and energy consumption.

## CONFLICT OF INTERESTS

The authors declare no conflict of interests associated with this publication.

## AUTHOR CONTRIBUTIONS

VL designed the study, conducted the experiment, analyzed and interpreted the data, and, drafted the manuscript. CM designed the study, interpreted data, and drafted the manuscript. AK conducted the experiments, interpreted the data, and edited the manuscript. ID interpreted the data, and edited the manuscript. All of the authors read, gave input, and approved the final manuscript.
